# Phase 1 trial of TPI 287, a microtubule stabilizing agent, in combination with bevacizumab in adults with recurrent glioblastoma

**DOI:** 10.1093/noajnl/vdae009

**Published:** 2024-01-18

**Authors:** Samuel A Goldlust, Louis B Nabors, Sigmund Hsu, Nimish Mohile, Paul J Duic, Tara Benkers, Samuel Singer, Mayank Rao, Lori Cappello, Sandra L Silberman, George Farmer

**Affiliations:** John Theurer Cancer Center, Hackensack University Medical Center, Hackensack, New Jersey, USA; Department of Neurology, University of Alabama at Birmingham, Birmingham, Alabama, USA; Mischer Neuroscience Institute, Memorial Hermann Health System, Houston, Texas, USA; Department of Neurology, University of Rochester Medical Center, Rochester, New York, USA; Long Island Brain Tumor Center at Neurological Surgery, P.C., Great Neck, New York, USA; Swedish Neuroscience Institute, Swedish Medical Center, Seattle, WA, USA; John Theurer Cancer Center, Hackensack University Medical Center, Hackensack, New Jersey, USA; Mischer Neuroscience Institute, Memorial Hermann Health System, Houston, Texas, USA; John Theurer Cancer Center, Hackensack University Medical Center, Hackensack, New Jersey, USA; Cortice Biosciences, New York, New York, USA; Cortice Biosciences, New York, New York, USA

**Keywords:** bevacizumab, clinical trial, glioblastoma, phase 1, taxane

## Abstract

**Background:**

Recurrent glioblastoma (rGBM) has limited treatment options. This phase 1 protocol was designed to study the safety and preliminary efficacy of TPI 287, a central nervous system penetrant microtubule stabilizer, in combination with bevacizumab (BEV) for the treatment of rGBM.

**Methods:**

GBM patients with up to 2 prior relapses without prior exposure to anti-angiogenic therapy were eligible. A standard 3 + 3 design was utilized to determine the maximum tolerated dose (MTD) of TPI 287. Cohorts received TPI 287 at 140–220 mg/m^2^ every 3 weeks and BEV 10 mg/kg every 2 weeks during 6-week cycles. An MRI was performed after each cycle, and treatment continued until progression as determined via response assessment in neuro-oncology criteria.

**Results:**

Twenty-four patients were enrolled at 6 centers. Treatment was generally well tolerated. Fatigue, myelosuppression, and peripheral neuropathy were the most common treatment emergent adverse events. Dose-limiting toxicity was not observed, thus the MTD was not determined. Twenty-three patients were evaluable for median and 6-month progression-free survival, which were 5.5 months (mo) and 40%, respectively. Median and 12-month overall survival were 13.4 mo and 64%, respectively. The optimal phase 2 dose was determined to be 200 mg/m^2^.

**Conclusions:**

TPI 287 can be safely combined with BEV for the treatment of rGBM and preliminary efficacy supports further investigation of this combination.

**Key Points**

- TPI 287 and bevacizumab were well tolerated in recurrent glioblastoma.
- Preliminary efficacy was encouraging.

**Importance of the Study**

Recurrent glioblastoma (GBM) has no established standard of care and limited treatment options. TPI 287 is unique among taxanes for its ability to evade blood-brain barrier efflux and readily accumulate in the brain following peripheral administration. This phase 1 study enrolled 24 patients with bevacizumab (BEV) naïve GBM and up to 2 prior relapses. Combination treatment with BEV and TPI 287 was well tolerated and efficacy compared favorably with historical data, demonstrating a median overall survival (mOS) of 13.4 months (mo). Among 8 patients with tumors of known unmethylated MGMT promoter, mOS was 11.6 mo, supporting the possibility of an effective treatment in a subset of patients with a poor prognosis and tumors unlikely to respond to alkylating chemotherapy. This data supports a phase 2 dose expansion study in the recurrent setting and an opportunity for evaluation in the frontline setting in lieu of temozolomide for patients with MGMT unmethylated tumors.

Glioblastoma (GBM) is the most common and aggressive primary brain tumor, with a U.S. incidence rate of 3 per 100,000 people and a median survival of only 8 months (mo) when all patients are considered, including those that opt against treatment.^1^ Recurrence following first-line treatment with adjuvant radiotherapy and temozolomide is nearly universal and there is no consensus standard of care at progression. Bevacizumab (BEV) carries an FDA-approved indication as monotherapy for recurrent GBM (rGBM), however, even among highly selected patients with GBM treated on clinical trials of BEV at first recurrence, median overall survival (mOS) only ranges 4–9 mo.^2–8^ Hence, there is a significant unmet need for new agents to treat this disease.

TPI 287 is a novel microtubule stabilizing agent of the taxane class of small molecules. Unlike other taxanes, including paclitaxel and docetaxel, TPI 287 is not a substrate for the P-glycoprotein molecular efflux pump. Consequently, as shown in preclinical studies, TPI 287 readily accumulates in the brain following intravenous administration.^9^ TPI 287 inhibits the polymerization of tubulin to the same degree as other taxanes, which results in microtubule stabilization in mitotic cells and cell death. Indeed, results have shown that TPI 287, but not paclitaxel, can prevent the formation of metastatic brain lesions in an aggressive mouse model of brain metastases.^9^ Moreover, in an orthotopic human GBM xenograft mouse model, TPI 287 in combination with the Aurora-kinase A (AURKA) inhibitor alisertib was shown to synergistically prolong survival and reduce tumor volume through induction of apoptosis.^10,11^ TPI-287 has been studied in over 350 patients to date, including clinical trials as monotherapy in hormone refractory prostate cancer, metastatic melanoma, and tauopathies, as well as in combination therapy with temozolomide for recurrent neuroblastoma and medulloblastoma. Evidence of activity was noted and the drug was generally well tolerated, with dose limitations related to neuropathy.^12–15^

Bevacizumab is a therapeutic monoclonal antibody designed to modulate tumor angiogenesis by inactivating vascular endothelial growth factor A (VEGF-A). Results of phase 3 randomized clinical trials in solid tumor malignancies have shown that the addition of BEV to chemotherapy (including taxanes) can improve progression-free (PFS) and overall survival (OS).^16^ Conversely, phase 3 trials of BEV in newly diagnosed and recurrent GBM have demonstrated improved PFS without OS benefit.^17–19^

Based upon the combinatorial activity of taxanes and BEV observed in other solid tumor indications, and the ability of TPI 287 to readily accumulate in the CNS, we designed a phase 1/2 clinical trial to evaluate the safety and efficacy of these drugs used in combination for rGBM. Results from the phase 1 portion of this study are reported here.

## Materials and Methods

### Key Eligibility Criteria

Adults (≥18 years) with rGBM, life expectancy of >12 weeks, KPS ≥ 70, and adequate marrow, renal, and liver function were eligible at recurrence. Up to 2 prior relapses were permitted, with criteria for determining progression per “Response Assessment Criteria for High Grade Gliomas” (RANO).^20^ Patients had to be at least 12 weeks post-completion of radiation unless there was unequivocal evidence of progression (ie, histologic confirmation or out-of-field recurrence), 6 weeks from nitrosoureas, 3 weeks from procarbazine, 4 weeks from experimental or other cytotoxic drugs, and 1 week from noncytotoxic agents (eg, interferon, tamoxifen, *cis*-retinoic acid). Key exclusion criteria included prior exposure to anti-angiogenic therapy, taxanes or vinca alkaloids, treatment with enzyme-inducing anti-epileptic drugs or strong inhibitors/inducers of cytochrome P450 3A4 or P450 2C8 within 2 weeks prior to protocol start, the presence of leptomeningeal tumor or gliomatosis cerebri, patients less than 4 weeks from major surgery, those with grade 2 or higher peripheral neuropathy, or those who received more than 1 course of radiation therapy or a cumulative dose in excess of 65 Gy.

### Treatment and Evaluations

The protocol was a phase 1/2 design, with the phase 1 stage based upon a standard 3 + 3 dose-escalation scheme. Safety was the primary endpoint with the objective of determining the maximum tolerated dose (MTD) of TPI 287 when administered in combination with BEV. Dose-limiting toxicity (DLT) was defined as any of the following adverse events (AE) occurring in the 1st cycle of treatment (1 cycle of 6 weeks defined as 2 infusions of TPI 287 every 3 weeks and 3 infusions of bevacizumab 10 mg/kg every 2 weeks): grade 4 myelosuppression, febrile neutropenia, grade 3 thrombocytopenia with bleeding, grade ≥3 transaminase elevation, grade ≥2 cardiotoxicity, any grade ≥3 nonhematologic toxicity (excluding nausea, vomiting, or diarrhea that resolved within 72 h), and grade 4 diarrhea or vomiting. Adverse events were assessed using the National Cancer Institute Common Terminology Criteria for Adverse Events (CTCAE) Version 4.0. The protocol and informed consent forms were approved by a centralized institutional review board. Informed consent was obtained from all patients. Determination of O^6^-methylguanine-DNA methyltransferase (MGMT) promoter methylation status was conducted retrospectively.

TPI 287 was provided in a 15:85 Kolliphor^®^ (BASF Pharma.): ethanol formulation diluted in saline and administered i.v. over 1 h. Dose cohorts included 140, 150, 160, 170, 180, 200, 220 mg/m^2^. Bevacizumab was administered i.v. at a fixed dose of 10 mg/kg. Patients were treated on days 1 and 22 with TPI 287 dosed according to treatment cohort and BEV on days 1, 15, and 29 of each 6-week cycle. Escalation to the next highest TPI 287 dose cohort did not occur until at least 3 patients completed the first cycle of treatment and qualified for safety assessment, per protocol. Patients deviating from this defined schedule during the first cycle were replaced and ineligible for DLT assessment but qualified for efficacy assessment if at least 2 doses of TPI 287 were administered. Magnetic resonance imaging and response assessment were conducted by the treating investigator per RANO criteria every 6 weeks, or sooner if disease progression was clinically suspected.^20^ Patients were followed for survival after coming off the study.

### Statistics

Overall survival was defined as the time from first treatment on protocol until death. Progression-free survival was defined as the time from first treatment on protocol until progression of disease or death. If a final event was not recorded, dates were censored as of the last radiographic analysis and/or confirmation of survival. Survival results were calculated using Kaplan–Meier methodology.

## Results

Between September 2013 and August 2015, a total of 24 patients at 6 U.S. institutions were enrolled, received at least 1 dose of TPI 287 and BEV, and were evaluable for safety. TPI 287 dosing began at 140 mg/m^2^ every 3 weeks in conjunction with BEV 10 mg/kg every 2 weeks. TPI 287 dose escalation to 150, 160, 170, 180, 200, and 220 mg/m^2^ occurred in a 3 + 3 fashion following protocol specified safety evaluations at each dose cohort. Twenty-three patients were evaluable for survival, per protocol. Demographics are provided in Table 1.

**Table 1.** Patient demographics (*n* = 24) and preliminary efficacy (*n* = 23)

Patient	Dose (mg/m^2^)	Age	Sex	KPS	MGMT Status	Tumor size (mm^2^)	PFS (months)	OS (months)
1	140	53	M	90	UNK	1036	5.6	12.9
2	140	41	F	100	UM	UME	2.8	12.2
3	140	52	M	90	UNK	384	5.5	17.9
4	150	51	F	100	UNK	UME	4.5	15.1
5	150	62	M	90	UM	481	11.3	18.2
6	150	56	M	90	UNK	1043	1.4	12.6
7	160	59	M	90	UM	UME	2.8	5.2
8	160	69	M	70	UNK	5311	N/A	N/A
9	160	50	M	90	UNK	560	10.7	21.9
10	160	75	F	90	UM	312	12.6	19.0
11	170	56	M	90	ME	UME	4.1	7.6
12	170	47	F	100	UNK	513	2.3	6.6
13	170	46	F	80	UNK	120	4.1	13.9
14	170	62	M	80	UNK	650	6.9	9.2
15	180	51	M	90	UM	1079	11.0	20.9
16	180	50	M	100	UM	180	4.1	10.9
17	180	66	M	70	UNK	2383	6.9	11.1
18	180	53	F	80	UNK	1353	1.4+	2.8+
19	200	56	F	90	UNK	UME	4.5+	19.4+
20	200	61	M	90	UNK	589	8.2	15.8
21	200	50	M	90	UNK	136	4.2	15.6
22	220	57	M	80	UM	506	4.1+	8.0
23	220	76	F	70	UM	963	2.7	6.9
24	220	68	M	90	UNK	UME	12.1	21.4

^*^ME = methylated, MGMT = O^6^-methylguanine-DNA-methyltransferase, OS = overall survival, PFS = progression-free survival, UM = unmethylated, UME = unmeasurable, UNK = unknown.

### Adverse Events

The combined treatment of TPI 287 and BEV was well tolerated (Table 2). Among the 24 patients evaluable for safety, no DLTs were reported and the MTD was not reached after dosing up to 220 mg/m^2^. As neuropathy was dose limiting in previous studies, escalation was halted upon the emergence of low-grade neuropathy in the present population. The most common treatment-related AE were fatigue (6 patients with grade 1/2, 1 report of grade 3), neutropenia (5 patients with grades 1/2, 2 patients with grade 3), peripheral sensory neuropathy (11 patients, all grade 1/2) and hypoesthesia (6 patients, all grade 1/2) which are consistent with AE reported with other taxanes.^21^ There were 4 reported serious adverse events, including 3 deemed unrelated to treatment on protocol, and 1 report of grade 3 neutropenia deemed related to TPI 287. No grade 3 or higher CNS-related AE attributed to drug treatment was reported, and ataxia was presumed to be related to peripheral nervous system dysfunction rather than cerebellar pathology. One case of grade 4 hypertriglyceridemia was observed without sequela, and no treatment-related deaths were reported.

**Table 2.** Treatment emergent adverse events (*n* = 24)

	Grade 1/2	Grade 3
Abdominal pain	2	
Alopecia	4	
Anal fistula		1
Arthralgia	3	
Ataxia	1	1
Back pain	3	
Confusion		1
Diarrhea	4	
Dysphasia		1
Eye pain	2	
Fatigue	6	1
Flushing	2	
Headache	3	
Hypertension		1
Lymphocyte count decreased	1	1
Memory impairment	2	
Myalgia	2	
Nasal congestion	2	
Nausea	4	
Neutrophil count decreased	5	2
Nervous system disorders—other, hypoesthesia	6	
Pain in extremity	2	
Peripheral sensory neuropathy	11	
Platelet count decreased	2	
Seizure	2	1
Skin and subcutaneous disorders—other, rash	2	
White blood cell decreased	4	

^*^One patient experienced grade 4 hypertriglyceridemia. All noted grade 1/2 adverse events (AE) were at least possibly related to TPI 287 and occurred in 2 or more patients. Grade 3/4 AE are reported regardless of frequency and relatedness to TPI 287 administration.

### Exploratory Efficacy Analysis

**Figure 1.**
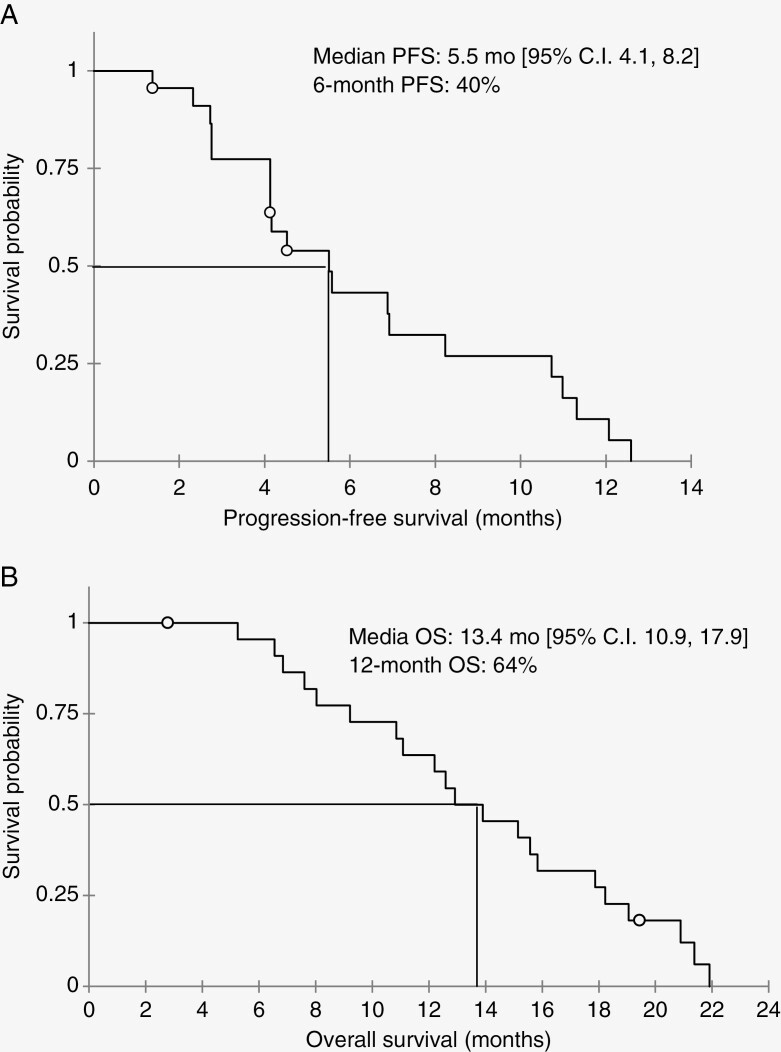
Progression-free survival (PFS) (A) and overall survival (OS) (B) of the 23 patients evaluable for efficacy treated with TPI 287 and bevacizumab.

MRI was conducted every 6 weeks or sooner for any clinical decline as deemed by the investigator. Per protocol 23/24 patients completed at least one 6 week treatment cycle and were evaluable for efficacy. After the occurrence of 20 progression events, median and 6-month PFS were 5.5 mo (95% CI 4.1, 8.2) and 40%, respectively. After the occurrence of 21 deaths, median and 12-month OS were 13.4 mo (95% CI 10.9, 17.9) and 64%, respectively (Figure 1). Of the 9 patients for whom tumor MGMT promoter methylation status was known, 8 (89%) harbored tumors with an unmethylated promoter. Median PFS and OS for this unmethylated subset were 7.5 mo (95% CI 2.8, 11.3) and 11.6 mo (95% CI 6.9, 19), respectively.

## Discussion

Effective drugs for the treatment of rGBM are limited. In 2010, BEV received accelerated FDA approval, becoming the first agent approved for this indication following lomustine decades earlier. The FDA later granted full approval to BEV for rGBM despite the fact that it failed to demonstrate an OS improvement in randomized trials for both newly diagnosed and rGBM.^17–19^

This phase 1 trial was designed primarily to determine the MTD of TPI 287 in combination with BEV for the treatment of rGBM. The most notable AE thought to be at least possibly related to TPI 287 treatment included fatigue, myelosuppression, and peripheral neuropathy, which are commonly associated with other taxanes. Dose escalation was halted at 220 mg/m^2^, prior to the determination of the MTD for increased frequency of low-grade peripheral neuropathy, leading 1 patient to withdraw consent. Importantly, this incident was considered grade 2 in severity and not dose limiting. Taking AE data from previous clinical trials of TPI 287 into consideration, toxicity in the present study that favored taxane over BEV, coupled with responses seen at lower doses, 200 mg/m^2^ was determined to be the optimal phase 2 dose as the consensus opinion of an independent clinical advisory board.

O^6^-methylguanine-DNA methyltransferase (MGMT) is a DNA repair enzyme implicated in GBM resistance to alkylating agents. Epigenetic silencing of MGMT via promoter methylation predicts response to temozolomide and nitrosoureas and may also be a positive prognosticator independent of treatment.^22–25^ While tumor MGMT promoter methylation data is incomplete for our trial population, 8/9 (89%) patients with known status were unmethylated. This suggests that the patients enrolled had a worse prognosis than the general GBM population, of which roughly 2/3 have unmethylated tumors. In this study, 3 of the 8 patients with unmethylated tumors had an OS exceeding 18 months. Although limited by the number of patients in this analysis, one would expect a microtubule inhibitor to exert cytotoxic impact independent of whether DNA repair pathways are intact. Accordingly, TPI 287, whose activity should be agnostic to MGMT expression, merits further study not only as a salvage regimen but also earlier in the disease course as an alternative to temozolomide in patients with unmethylated tumors.

Beyond incomplete molecular data including MGMT promoter methylation and isocitrate dehydrogenase (IDH) mutation status, other limitations of the present study include a small sample size, and absent central pathology and imaging review, with a plan to address these deficiencies in phase 2. Following the conception of the protocol, the field of neuro-oncology moved towards single-agent trials in IDH wild-type tumors based upon revised WHO 2021 criteria to clearly discern signals of benefit in a uniform population.^26^ There was also a delay in publishing the data, which was ultimately initiated by the investigators due to lack of progress by the sponsor related to personnel turnover.

As a phase 1 study, the primary goal was to establish safety. Although preliminary efficacy is reported, this awaits confirmation in phase 2. Given that there remains no consensus opinion for the treatment of rGBM, careful consideration of the inclusion of BEV in the phase 2 dose expansion is warranted, with the understanding that response would not be a meaningful outcome measure. Lastly, the incorporation of a window of opportunity/ phase 0 arm for patients who are surgical candidates as well as a more comprehensive approach to serial laboratory analysis in phase 2 will offer an improved understanding of the pharmacokinetics and pharmacodynamics of TPI 287.

## Data Availability

Data generated in this study will be made available upon reasonable request by contacting the corresponding author.
